# A Uniform Energy Consumption Algorithm for Wireless Sensor and Actuator Networks Based on Dynamic Polling Point Selection

**DOI:** 10.3390/s140100095

**Published:** 2013-12-19

**Authors:** Shuo Li, Jun Peng, Weirong Liu, Zhengfa Zhu, Kuo-Chi Lin

**Affiliations:** 1 School of Information Science and Engineering, Central South University, Changsha 410075, China; E-Mails: lishuo@csu.edu.cn (S.L.); weirong_liu@126.com (W.L.); jws206@163.com (Z.Z.); 2 Department of Mechanical and Aerospace Engineering, University of Central Florida, Orlando, FL 32816, USA; E-Mail: kurt.lin@ucf.edu

**Keywords:** wireless sensor and actor networks, mobile data collection, network lifetime, uniform energy consumption

## Abstract

Recent research has indicated that using the mobility of the actuator in wireless sensor and actuator networks (WSANs) to achieve mobile data collection can greatly increase the sensor network lifetime. However, mobile data collection may result in unacceptable collection delays in the network if the path of the actuator is too long. Because real-time network applications require meeting data collection delay constraints, planning the path of the actuator is a very important issue to balance the prolongation of the network lifetime and the reduction of the data collection delay. In this paper, a multi-hop routing mobile data collection algorithm is proposed based on dynamic polling point selection with delay constraints to address this issue. The algorithm can actively update the selection of the actuator's polling points according to the sensor nodes' residual energies and their locations while also considering the collection delay constraint. It also dynamically constructs the multi-hop routing trees rooted by these polling points to balance the sensor node energy consumption and the extension of the network lifetime. The effectiveness of the algorithm is validated by simulation.

## 1. Introduction

A wireless sensor network is composed of a number of collectors and many low-cost, resource-limited sensor nodes. Sensor nodes are distributed in the region of interest, collect sensor data from that region and, then, forward those data to a remote data sink for environmental monitoring, military surveillance, fire detection, animal tracking or other applications. Because it is difficult to replace or recharge sensor node batteries while the sensor node is in service, one of the main concerns of a wireless sensor network is to increase its energy efficiency.

In traditional wireless sensor networks, the locations of sensor nodes and data sinks are fixed once they have been distributed, and the data created by the sensors are forwarded to the sinks by a multi-hop relay. Network efficiency is increased by optimizing the scheduling policy, aggregate routing [1] and sensor node load balancing [2], but a multiple hop relay will inevitably result in high energy consumption during data transmission.

In wireless sensor actuator networks, mobile data gathering is achieved by the mobility of the actuator and unlimited hardware resources to reduce energy consumption. During each data gathering period, the actuator starts from the sink, travels through the entire network and collects the data from nearby sensor nodes while in motion, before returning to forward its collected data to the sink. In ideal circumstances, the actuator's moving distance is not limited. It is able to visit all of the sensor nodes in the network in order, communicating with the sensor nodes by single-hop relay, thus minimizing energy consumption during communication. However, in practical applications, strict restrictions are placed on the data collection delay. Thus, the key issue of using actuators in wireless sensor networks is planning reasonable paths for the actuator and optimizing the data exchange mechanisms with the sensor nodes.

Further research indicates that actuators increase the energy efficiency of wireless networks by reducing the number of relay hops within the network. However, the sensor nodes close to the polling points still require the transmission of more data packets whose energies expire quickly, leading to non-uniform energy consumption and restricting the network's lifetime. With meeting the network convergence delay requirements as a prerequisite, this paper aims to increase the network lifetime by proposing a multi-hop routing mobile data collecting algorithm based on dynamic polling point selection under delay constraints. The dynamic selection of polling points will improve the network's energy efficiency and extend the network lifetime as much as possible; multi-hop communications and an optimized actuator moving path will guarantee the network data collection delay.

The rest of this paper is organized as follows. In Section 2, related works are reviewed. In Section 3, assumptions concerning the integer linear programming (ILP) problem and its formulation are discussed. In Section 4, a uniform energy consumption algorithm is introduced. In Section 5, the comparative performance evaluation and simulation results are shown. Finally, the conclusions are drawn in Section 6.

## 2. Related Work

The issue of energy efficiency has been extensively studied in static wireless sensor networks. Those works have mostly focused on energy conservation or the balancing of energy consumption. The methods suggested to reduce network energy consumption include one or more of the following: topology controls, transmission power control, sensor node scheduling, coverage control, clustering and energy efficient routing.

Recent works have exploited the availability of the controlled mobile actuators to balance the energy consumption of sensor nodes. Based on the mobile actuator's transmission hop numbers, the existing research works are classified into two categories: single hop and multiple hops. In the first category, the mobile actuators only collect data from sources within a single hop. In [3], Shah *et al*. use mobile MULEs to collect data via random walks. This method leads to substantial power savings at the sensor nodes, as they only have to transmit over a short range. However, the cost is a higher data collection latency. Random walks cannot be optimized or guarantee the arrival of urgent messages within a time constraint. To overcome this problem, Gu *et al*. [4] proposes a heuristic solution, called earliest deadline first (EDF), which uses two variables to guide the mobile mules' motions. Recently, to achieve better scalability, a longer network lifetime and lower data collection latency, Zhao *et al*. uses multi-input multi-output (MIMO) and space division multiple access (SDMA) techniques to upload data to a mobile collector in [5,6]. In [5], the framework employs distributed load-balance single-hop clustering and multiple cluster heads in each cluster to balance the workload and facilitate the MIMO data uploading. Zhao *et al*. [6] extends a similar framework to multiple mobile collectors and proposes three heuristic algorithms: the maximum compatible pair (MCP), minimum covering spanning tree (MCST) and revenue-based (RB) algorithms. While those works minimize the energy cost and balance energy consumption by avoiding multi-hop relays, they may also result in long data collection latency when the network scale becomes larger.

The second category allows the mobile actuators to collect data via multi-hop routings. The maximum amount shortest path (MASP) data collection strategy proposed by Gao *et al*. [7] is for mobile equipment moving along a constrained path. The sensor nodes within a one-hop distance from the mobile equipment are elected as the proxies. The proxies collect data from the rest of the network through multi-hop routings. Konstantopoulos *et al*. [8] introduce MobiCluster, a protocol that uses urban buses to carry mobile stations that retrieve information from isolated parts of WSNs. MobiCluster mainly aims to maximize the connectivity and data throughput and to enable the energy expenditure balance among sensor nodes. The mobile elements are all moving along fixed paths in [7,8]. Considering the scenario in which the sink node moves at a high speed, Oliveira *et al*. [9] propose the Whisper (Wireless High Speed Routing) algorithms for routing data towards the sink node's current position or even toward a future position. When the actuators can move freely in the network, it becomes important to decide the sojourn positions for the mobile elements. In such a scenario, Luo and Hubanx build a framework for investigating the joint actuator mobility and routing problem by constraining the actuator to a finite number of locations to prolong the network lifetime in [10,11]. Gatzianas and Georgiadis [12] optimize the network lifetime by formulating a linear programming problem that incorporates the actuator sojourn times and the routing flow vector for each actuator location.

Utilizing multiple actuators can reduce the network energy consumption further and also improve the data delivery ratio [13–15]. In [13], the controlled and coordinated multiple actuators are deployed to improve the lifetime of the WSN. It defines a centralized heuristic to determine the routes and sojourn times for the controlled actuator mobility and then defines a distributed protocol for the coordinated actuator movements based on the expected lifetime improvements produced by an actuator moving to a new site. Erman *et al*. [14] present a data dissemination protocol based on a virtual infrastructure called Honeycomb Architecture to deliver an emergency message from static sensor nodes to the mobile sinks. Once a query reaches the central hexagon, the reply is sent in the reverse routing path. A bio-inspired networking cooperation scheme among wirelessly connected static and mobile sensor nodes is proposed by Freitas *et al*. [15]. This proposed scheme does not need any route discovery and pre-known information about the mobile nodes. Using pheromone-based communication, the message can be quickly delivered from static nodes to sink nodes.

Those approaches can effectively reduce the energy consumption and extend the network lifetime. However, they do not impose any constraint on the data latency caused by the actuator's mobility. In [16], Keung *et al*. study the message delivery capacity problem considering three fundamental factors: the buffer size, the delay constraint and the message relay policy. The object is to maximize the message delivery capacity subject to the delay and buffering constraints. Xing *et al*. [17] propose a rendezvous design to minimize the distances of the multi-hop routing paths based on the minimum Steiner tree under a delay constraint. Zhao and Yang [18] explore the balance between latency and energy consumption in mobile data gathering by exploring the tradeoff between the relay hop count of the sensor nodes for local data aggregation and the tour length of the mobile collector.

Further research shows that the sensor nodes adjacent to the actuator's current polled position still need to forward more data packages. Therefore, those sensor nodes exhaust their energy earlier than other sensor nodes, which results in non-uniform energy consumption and constraints on the network lifetime. To address this problem, this paper proposes a uniform energy consumption algorithm for wireless sensor and actuator network (WSAN) based on dynamic polling point selection.

The algorithm must address two key problems: (1) how to generate the shortest path tree of the network; and (2) how to select the polling points and drive actuator movements to meet the constraints on data collection delay. In particular, to balance the energy consumption, the sensor node's residual energy should be incorporated into the algorithms, so that the path tree and actuator movement can be updated dynamically.

## 3. Problem Description and Formulation

In this section, the definitions of the network lifetime, in-degree and energy consumption model are given. We then present the system network topology using graph-theoretical methods. We assume that the system network topology of WSAN (excluding the actuators and the sink) is a tree structure, which has been widely discussed by previous works. At the same time, several assumptions of the network are proposed as basic conditions. We then propose a energy consumption model for each component of the network and derive the cost between neighboring sensor nodes. Accordingly, an integer linear programming (ILP) problem, called the energy efficient relay, and a sink routing problem are formulated, and the definition of the problem is given.

### 3.1. Essential Conception and Definition

#### 3.1.1. Network Lifetime

From the literature [10,19,20], there are several general methods to define the network lifetime. Luo *et al*. [10] defines the network lifetime as the time span from the sensor node deployment to the first loss of coverage. In [19], using simple and intuitive methods, the minimum system lifetime is defined as the operational time of the local cluster until the first sensor node in the cluster runs out of power. In [20], the break probability is introduced to illustrate the definition of the network lifetime in both stochastic and deterministic manners. In this paper, we define the network lifetime as the operational time of the network until the first sensor node in the network runs out of power.

#### 3.1.2. In-Degree

The in-degree is one of the most essential concepts of the graph theory. There is a good deal of literature illustrating the concept of the in-degree. The definition of the in-degree is illustrated below:

Definition 1: For the case of undirected graph, *G* (*V*, *E*), with vertices set *V* = {*υ*_1_,*υ*_2_,…,*υ_n_*} and edges set *E* = {〈*υ_i_*, *υ_j_*〉, 〈*υ_j_*, *υ_i_*〉 : ∀*υ_i_*, *υ_j_* ∈ *V*,*i* ≠ *j*}, *Inc*(*υ_i_*) is defined as *Inc*(*υ_i_*) ≜ {*e_ij_*|*e_ij_* = 〈*υ_i_*, *υ_j_*〉 ∈ *E*}, where *Inc*(*υ_i_*) denotes the set of edges related to vertex *υ_i_*. For the case of the directed graph, *G* (*V*, *E*), *Inc*(*υ_i_*) is defined as follows:

$$\begin{array}{ll} Inc^{+}(\upsilon_{i}) & ≜\{e_{ij}|e_{ij}=\langle \upsilon_{i},\upsilon_{j}\rangle \in E\}\\ Inc^{-}(\upsilon_{i}) & ≜\{e_{ij}|e_{ij}=\langle \upsilon_{j},\upsilon_{i}\rangle \in E\} \end{array}$$

where *Inc*^+^(*υ_i_*) denotes the set of edges whose starting point is *υ_i_* and *Inc*^−^(*υ_i_*) denotes the set of edges whose end point is *υ_i_*. Accordingly, the out-degree of vertex *υ_i_* is defined as *d*^+^(*υ_i_*) ≜ |*Inc*^+^(*υ_i_*)|, and the in-degree of vertex *υ_i_* is defined as *d*^−^(*υ_i_*) ≜ |*Inc*^−^ (*υ_i_*)|. Further, *d*(*υ_i_*) ≜ *d*^+^(*υ_i_*) + *d*^−^(*υ_i_*) denotes the degree of vertex *υ_i_*.

#### 3.1.3. Energy Consumption Model

A sensor node is composed of the sensors, processing unit, memory, RFtransceiver and battery. The total energy consumption by the sensor node can be expressed as the sum of the energy consumption by each element [21]:

(1) $$E_{\text{node}}=E_{\text{sensor}}+E_{\mu c}+E_{\text{trans}}+E_{\text{recv}}$$

First, the energy consumption by the sensor is expressed as follows:

(2) $$E_{\text{sensor}}=P_{\text{sensor}}(t_{\text{stabilization}}+t_{\text{measure}})$$

where *t_stabilization_* corresponds to the stabilization time of the sensor and *t_measure_* to the duration of the sensing phase, depending on the number of measures. Additionally, *P_sensor_* is the power consumption of the sensor. After the sensing phase, the microcontroller proceeds to data processing, formatting and coding, in accordance with the application and communication protocols. The energy consumption during the data processing phase is expressed as follows:

(3) $$E_{\text{data}\_\text{process}}=\frac{N_{\text{soft}}}{S_{\mu c}}P_{\mu c}$$

where *N_soft_* indicates the number of instructions per cycle according to the embedded software, *S_μc_* is the microcontroller speed and *P_μc_* is the power consumption of the microcontroller.

After that, the data transmission depends on the goal of the application. In some cases, it is possible to aggregate several measurements before sending data. In other applications, the data are sent only when an event is detected. Moreover, in many cases, a receiver is needed to acknowledge the sender, to respond to the data sink request and to relay data from another sensor node. The energy consumption of the transmitter and receiver is defined as, respectively:

(4) $$E_{\text{trans}}=\frac{N_{\text{bits}\_\text{trans}}}{D_{\text{inst}}}P_{\text{trans}}$$

(5) $$E_{\text{recv}}=\frac{N_{\text{bits}\_\text{recv}}}{D_{\text{inst}}}P_{\text{recv}}$$

where *N_bits_trans_* and *N_bits_recv_* are the number of bits to transmit and to receive, respectively. *D_inst_* is the instantaneous data rate. *P_trans_* and *P_recv_* are the power consumption of the transmitter and receiver.

Finally, the consumption of the microcontroller is expressed as follows:

(6) $$E_{\mu c}=P_{\mu c}(t_{\text{sensor}}+\frac{N_{\text{soft}}}{S_{\mu c}}+t_{\text{trans}}+t_{\text{recv}})$$

in which the time parameters of the microcontroller may be taken as constant, depending on the application requirements and the MACprotocol of the network. In fact, the role of the microcontroller is to manage the different operating modes of the sensor node: measuring, processing, transmitting and receiving.

### 3.2. Problem Formulation

The network topology of the WSANs is modeled as a directed graph, *G* (*V*, *E*), where *V* = {*υ*_1_, *υ*_2_, …, *υ_n_*} is the set of all static sensor nodes and *E* = {〈*υ_i_*, *υ_j_*〉 : ∀*υ_i_*, *υ_j_* ∈ *V*, *i* ≠ *j*} is the set of all directed links. There is a cost, *C_ij_*, assigned to each link. In addition to the static sensor nodes, there is a set *κ* = (1, …, *k*) of actuators that collect data from the sensor nodes; we assume that *k* ≪ *n*. There is only one sink node in the WSANs, which is the data aggregation point.

There are several assumptions for the WSAN:

(i) The sensor nodes are stationary and distributed in a two-dimensional region. All the sensor nodes have the same transmission power and initial energy. Additionally, these sensor nodes operate in the duty cycle mechanism [15,22]. In one cycle, the sensor node takes some time to listen and transmit data, called the active state; while during the cycle's rest time, the sensor node can turn off almost all its devices, called the sleep state, in order to save energy. A duty cycle mechanism defines sensor node periods and their active time windows. In the paper, it is assumed that the active sensor nodes' density is enough to cover the sensed region, and the sensor nodes' active time window size is larger than the data collection time constraints in order to to guarantee sensor nodes' activity during a single data collection cycle of the actuator.
(ii) The actuators have no energy limit and can move freely throughout the region to collect data and then upload to the sink; when the actuator collects data, the sensor node will send its active time window, attaching to the sensor data packets to polling points, which then relay these data to the actuator, so that the actuator can update the sensor node state and select an active sensor node set properly.
(iii) Certain sensor nodes are chosen as polling points, which aggregate the data from sensor nodes and deliver them to the actuator. The actuator will visit those polling points one by one and, finally, return to the sink.
(iv) The data traffic originates from each sensor node with a fixed generation rate and flows to one of the polling points within a single-hop or multi-hops. Polling points have sufficient storage capacity to buffer the total volume of data generated by the sensor nodes within delivery deadline *D*.
(v) The actuator moves with a constant speed, *s*, and the data collection delay is mostly caused by the moving time of the actuator. Therefore, the collection deadline is related to the maximum length of the actuator's tour by *L* = *sD*.
(vi) The sensor nodes and actuators are assumed to know their own physical locations through the GPS or a locations service in the network.
(vii) Sensing, information processing and data transmitting and receiving are three factors in the energy consumption of a sensor node.

Assumptions (i) and (ii) guarantee that the best possible energy saving and balancing under the time-constraint can be achieved; an actuator's tour is defined by assumption (iii); that is, the location of the polling points will be the sojourn points of the actuators. Nesamony *et al*. [23] considers that the actuators can sojourn at the edge of each polling point's communication range modeled by a circle. It is obvious that the communication range of a sensor node is much smaller than the tour length of the actuators when the network scale becomes large. Multi-hop communication is used to shorten the tour length of the actuators and map the deadline to a maximum tour length, as shown in assumptions (iv) and (v); assumption (vi) is realistic, because many WSANs need to gather spatially distributed information about the environment. Finally, assumption (vii) means that the energy consumptions for sensing, local aggregation and processing are negligible for the energy model.

In addition, we ignore the influence of transmission interference between relevant sensor nodes and data error during transmission, so some additional assumptions are proposed as follows: (1) end-to-end data transmission is assumed to be reliable; and (2) radio interference can be avoided by a multiplex mechanism, such as frequency-division multiplexing (FDM), time-division multiplexing (TDM) or code-division multiplexing (CDM).

In this paper, the polling-based, multi-hop mobile data collection scheme can be formulated into an optimization problem, called the energy efficient relay and moving path-planning problem. The problem clearly consists of two sub-problems. The first is the energy minimization path-planning problem. The second is the energy balancing load assignment problem. The two problems can be formulated as the following integer linear programming (ILP) problem, which is formulated by Equations (7–19).

Its optimal objective is represented by Equations (7) and (8), which is based on reference [18] with a variation to include the multi-actuator energy balance requirement. Equation (7) is the network energy minimum objective, and Equation (8) reflects the energy balance among multi-actuators.

(7) $$\text{minimize}\sum_{(i,j)\in V,i\neq j}\left|C_{ij}\right|$$

(8) $$\sum_{(i,j)\in A_{1},i\neq j}\left|C_{ij}\right|=\sum_{(i,j)\in A_{2},i\neq j}\left|C_{ij}\right|=\ldots =\sum_{(i,j)\in A_{k},i\neq j}\left|C_{ij}\right|$$

There are key constraints that must be considered for practical data collection applications. The constraints should be comprised of aspects, such as: the polling point selection, the data packet routing, avoiding repeated loop visits and the moving length and total energy constraints. The following Equations (9–19) describe these constraints. Equation (9) ensures that a sensor node should deliver its own data to one and only one polling point.

(9) $$\begin{array}{lll} s.t. & \sum_{u\in V}y_{iu}=1, & \forall i\in V \end{array}$$

Equation (10) allows a polling point to have more than one sensor node (including itself).

(10) $$\begin{array}{ll} \sum_{i\in V}y_{iu}\geq I_{u}, & \forall u\in V \end{array}$$

In addition, Equation (11) establishes the restriction that only the polling point can be the root of aggregate tree.

(11) $$\begin{array}{ll} y_{iu}\leq I_{u}, & \forall i,u\in V \end{array}$$

The above Equations (9–11) give the properties of polling point selection. Equation (12) enforces that each sensor node should only deliver data to the polling point.

(12) $$\begin{array}{ll} x_{\text{iju}}\leq I_{u}, & \forall i,j,u\in V \end{array}$$

Equation (13) requires that only the neighboring sensor node affiliated with the same polling point can be the forward sensor node.

(13) $$\begin{array}{ll} x_{\text{iju}}\leq 0.5(y_{iu}+y_{ju})n_{ij}, & \forall i,j,u\in V \end{array}$$

Equation (14) forbids circular routs in the tree.

(14) $$\begin{array}{ll} \sum_{i,j\in V,i\neq j}x_{\text{iju}}=\sum_{i\in V,i\neq u}y_{iu}, & \forall u\in V \end{array}$$

The above Equations (12–14) dictate the routing between the sensor nodes and polling points. Equation (15) enforces that no polling point can be visited by more than one actuator at the same time.

(15) $$\begin{array}{ll} \sum_{k\in \kappa }z_{uk}\leq I_{u}, & \forall u\in V \end{array}$$

Equations (16) and (17) ensure that every polling point can only be visited once by an actuator.

(16) $$\begin{array}{ll} \sum_{p\in V}e_{\text{pqk}}=z_{qk}, & \forall q\in V,\forall k\in \kappa \end{array}$$

(17) $$\begin{array}{ll} \sum_{q\in V}e_{\text{pqk}}=z_{pk}, & \forall p\in V,\forall k\in \kappa \end{array}$$

The actuator moving length constraint and the total energy constraint are given in Equations (18) and (19), respectively.

(18) $$\begin{array}{ll} \sum_{p,q\in V,p\neq q}l_{pq}e_{\text{pqk}}\leq sD, & \forall k\in \kappa \end{array}$$

(19) $$\sum_{i\in V}\sum_{j\in V\backslash i}C_{ij}y_{ij}\leq |V|E_{\text{init}}$$

Equations (7–19) represent the data collection problem transformed to an integer linear programming problem for large-scale networks. Table 1 gives its variables definitions and meanings. The ILP problem includes the network energy minimum and balance requirement and reflects the tradeoff between energy consumption and collecting delay.

## 4. In-Degree Priority Algorithm

The objective of this paper is to solve the two sub-problems: the energy balancing load assignment problem and the energy minimization path-planning problem. Due to the constraints of the ILP problem, the two sub-problems can be translated into the following issues: (1) the generation of the shortest path tree of the network; and (2) the selection of the polling points and the strategy for actuator movement.

In this section, we first discuss how to generate the shortest path tree with the cost between neighboring active sensor nodes in order to guarantee uniform energy consumption. Then, to solve the energy minimization path-planning problem, the algorithm for polling point selection and the strategies for actuator movement are presented. According to the above algorithms, a new algorithm, the in-degree priority algorithm (IPA), is introduced, which considers not only the distance between the neighboring sensor nodes, but also their residual energy. Finally, the case of multiple actuators is discussed by expanding IPA to the multiple actuator uniform energy consumption problem, called the in-degree priority algorithm for multiple mobile actuator (IPA-MMA).

### 4.1. Dynamic Shortest Path Tree

In WSAN, the sensor nodes gather information and transmit data to the sink through single-hop or multi-hop communication. Thus, it is necessary to generate a tree with a root node that is the active sensor node nearest to the sink and with leaf nodes that are the other active sensor nodes transmitting data to the root node directly or via polling points. There are some works in the literature discussing how to create a shortest path tree (SPT). Xing *et al*. [17] propose a minimum Steiner tree, and Zhao *et al*. [18] use a shortest path tree to solve such a similarly complex problem. However, they all regard the tree as a static one and assume the tree to be constructed solely by the distance of the neighbor sensor nodes, which is called the cost between the neighboring sensor nodes.

**Figure 1.**
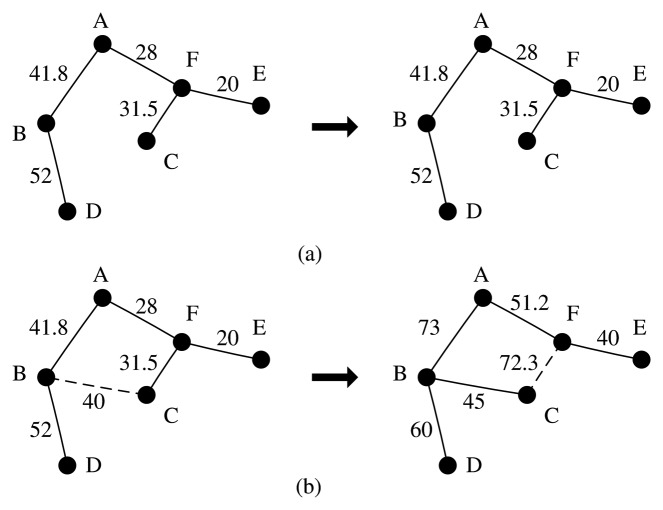
(**a**) Static shortest path tree, with the Euclidean distance between sensor nodes as the edge weight; (**b**) dynamic shortest path tree, with the Euclidean distance between the sensor nodes and the residual energy as the edge weight.

In this study, a definition of link cost *C_ij_* is introduced that considers not only the factor of distance between the neighboring sensor nodes, but also their residual energy. The link cost, *C_ij_*, is defined as follows:

(20) $$C_{ij}=DST_{ij}/(r_{i}\times r_{j})$$

where *DST_ij_* is the Euclidean distance between sensor nodes, *r_i_* is the normalized residual energy of sensor node *i* and *r_j_* is the normalized residual energy of sensor node *j*. In this work, the link cost, *C_ij_*, is set as the weight of an edge to construct the dynamic shortest path tree. As the sensor nodes' residual energies change dynamically, the link cost, *C_ij_*, also changes accordingly; that is, a dynamic shortest path tree is constructed to balance the energy consumption of the network. It is true that a sensor node will consume more energy with a heavier load, so the link cost, *C_ij_*, will also increase. When we construct the shortest path tree with Equation (20), those links associated with the heaviest sensor node have a larger cost, so we will choose links with a lower cost. An example is shown in Figure 1.

### 4.2. Polling Point Selection and Actuator Moving Strategy

The energy consumption of a sensor node is related to two factors, the in-degree of the sensor node and the distance to its neighboring sensor nodes. Thus, the higher the in-degree and the longer the distance, the more easily the energy of the sensor node is exhausted. Based on these principles, a method of polling point selection and a strategy of actuator movement are proposed. The actuator movement strategy is actually to find the shortest round trip among the polling points and the sink, which is exactly the traveling salesman problem (TSP). We run the nearest neighbor algorithm [24] for the TSP problem. The function, *TSP*(*P*), can calculate the shortest moving distance of the actuator visiting the selected polling points in *P*, where *P* is the set of polling points.

However, the actuator does not have to go to the exact locations of the polling points to receive data, because the polling points have a communication range and can transmit the data to the actuator within the communication range. The communication range can be incorporated into the algorithm to further improve the efficient utilization of energy, so the location of polling points would be modified to a new nearby location according to the communication range of the polling points. In the paper, the modified location could be calculated by the “runtrack” algorithm to reduce the average hop number further [25].

First, a dynamic shortest path tree of the network is constructed as in Section 4.1, denoted by *T*, connecting all sensor nodes in the network. The root of the tree is the sensor node closest to the sink. The Dijkstra algorithm can be used to construct such a tree. Next, the sensor node with the largest in-degree in the tree will be put into the temporary polling point set. If there are several sensor nodes with the same in-degree, the sensor node closest to the root will be chosen. A heuristic algorithm called “runtrack” is utilized to re-calculate the new locations of the polling points. Then, the actuator traverses the tree, *T*, from the source (in the first iteration, the source is the sink node) to the chosen sensor node until the moving distance of the actuator reaches *L*, where *L* is the maximum distance that the actuator can move in a round.

The covered sub-tree, denoted by *T′*, is recursively expanded until the moving distance of the actuator exceeds *L* or all the sensor nodes in the network have been chosen as polling points. The polling point set, *P*, is iteratively increased at the same time. In each iteration, the sensor node with the largest in-degree in *T*\*T′* will be selected as a polling point and added to *P*. The termination condition of the iteration is *L* − *D_a_* < *σ*, where *D_a_* is the moving distance of actuator and *σ* is a small constant to balance the solution quality and the time complexity.

**Figure 2.**
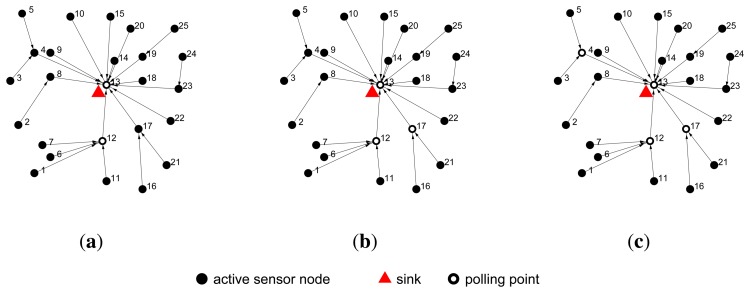
The polling point selection of in-degree priority algorithm (IPA). The black nodes represent the active sensor nodes; the triangle represents the sink, and the hollow nodes represent the polling points selected by the algorithm. IPA iterates four times and selects four polling points.

An example is given in Figure 2, where 25 active sensor nodes are scattered over a field with the sink (red triangle) located in the center of the area. The algorithm is executed by the actuator. In the initial period, sensor node 13 is taken as the root of our shortest path tree, because it is the closest sensor node to the sink. The actuator then constructs the tree, *T*, as in Section 4.1, and the tree is shown in Figure 2. In the first iteration, as Figure 2a shows, sensor node 12 is found as the largest in-degree sensor node on *T* with an in-degree of four; it is selected as the polling point in this iteration. In the second iteration, as Figure 2b shows, the in-degree of sensor node 4 and node 17 are both two, but sensor node 17 is closer to the sink, so it is selected as the polling point. In the third iteration, as Figure 2c shows, sensor node 4 is selected as the polling point. Once a sensor node is selected as the polling point, it will be deleted from *V* and added to *P*.

**Figure 3.**
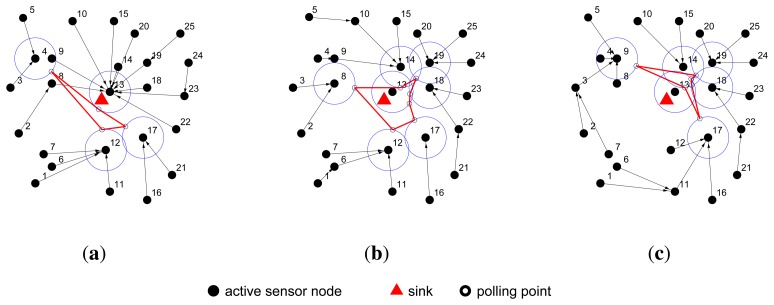
The dynamic moving path planning of IPA. The edges of the red polygons represent the moving path of the mobile actuator, and the blue circles represent the communication range of the polling points. IPA periodically reconstructs the shortest path tree and then re-selects the polling points before dynamically adjusting the path planning of the actuator.

In Figure 3, the moving path of the actuator is highlighted by the red line, and the blue circles indicate the communication range of the polling points. This group of figures shows that after several rounds, as the *C_ij_* values of every edge (*υ_i_*, *υ_j_*) change, the shortest path tree is reconstructed and the moving path of the actuator is also changed.

### 4.3. In-Degree Priority Algorithm

In this research, an in-degree priority algorithm (IPA) is introduced, which is comprised of two parts: (1) the generation of a shortest path tree with cost *C_ij_*; and (2) the selection of the polling points and the strategies for actuator movement. Algorithm 1 shows the process of IPA working in the network. *R* denotes the communication range of sensor nodes; *R_a_* is the communication range of the actuator, and *subt_u_* is the sub-tree rooted at sensor node *u*. We call each iteration of the algorithm a “round”.



**Algorithm 1** In-degree priority algorithm (IPA).
**Input:** *V*, *L*, *G*, *R*, *R_a_*, *σ*;**Output:** RNlist *P′*, a set of geometric trees *T′* = {*subt_u_*|*u* ∈ *P*}, and the path, *U*;1:Select the sensor nodes that are active in the next actuator tour in order to construct the active sensor node set;2:Calculate *C_ij_* for every edge (*υ_i_*, *υ_j_*) ∈ *E*. Set *C_ij_* as the weight of an edge, and then construct SPT tree *T*, which connects all points in *V*;3:*P* = 0; *P′* = 0; *U* = 0; *D_a_* = 0; *T′* = 0;4:**while** (*X* = *L* − *D_a_* > *σ*)5: Find the sensor node, *p_i_*, with the largest in-degree and closest to the sink in *V \ P*;6: *P* = {*p_i_* | *p_i_* is the sensor node with the largest in-degree in *P*};7: *P′* = *CR*(*P*, *R_a_*);8: Find the shortest path, *U*, visiting *P′* using the function, *TSP*(*P′*) ;9: Calculate the moving distance, *D_tsp_*, of path *U*;10: *D_a_* = *D_a_* +*D_tsp_*;11: Denote the sub-tree rooted at *p_i_* as *T′*;12:**end while**13:Find a set, *T′*, and an approximate shortest path, *U*, visiting *P′*;


There are two functions in the IPA algorithm. One is *TSP*(*P*), which uses the nearest neighbor algorithm to calculate the shortest moving distance of the actuator visiting the selected polling points in *P. P* is the set of polling points. The other is *CR*(*P*, *R_a_*), which would determine the new polling points, *P′*, based on the polling points when considering the communication range denoted by *R_a_* in the IPA algorithm.

The time complexity of IPA has three parts: the construction of a shortest path tree, the selection of polling points and the traveling salesman problem. We assume that there are a total of *N* sensors nodes distributed in the network. It takes *O*(*N*^2^) time to find the shortest path tree using the Dijkstra algorithm. The actuator's moving length is expanded by approximately half of the moving length in the last iteration, and one TSP tour is computed in the current iteration. The number of iterations is in the order of *logL*, and it takes at most *O*(*N*) time to generate an approximate shortest tour on all polling points if we use the nearest neighbor algorithm. Thus, the total time of IPA is *O*(*m*(*N*^2^ + *NlogL*)), in which *m* is the number of iterations of the IPA.

By analyzing the spatial complexity of the IPA algorithm, its memory requirements can be estimated. In the IPA algorithm, the input parameters and temporal variables include the node coordinates, the in-degree, the residual energy and the address, so the space complexity is *O*(5*N*). The output parameters include the adjacent matrix and the polling point queue, so the space complexity is *O*(*N*^2^ + *N*). The total space complexity is *O*(*N*^2^ + 6*N*).

### 4.4. IPA-MMA

Now, IPA is expanded to the multiple actuator uniform energy consumption problem, called IPA-MMA. IPA-MMA is based on the algorithm proposed by Zhao *et al*. [6]. As in the first step of IPA, IPA-MMA constructs the shortest path tree using Equation (20). Then, the algorithm assigns each sensor node *i* a weight, *w*(*i*), which includes the residual energy and the distance to its child nodes, calculated according to the following criteria:

(21) $$w(i)=\sum_{j\in V(\text{sub}(T(i)))}(\xi_{j}-r_{j})+\sum_{e\in E(\text{sub}(T(i)))}\eta \zeta_{e}$$

where *η* is a constant that represents the energy consumption of a link to deliver a packet for a unit length, *ξ_j_* represents the initial energy of the sensor nodes and *r_j_* represents the residual energy of the sensor nodes. The *sub*(*T*(*i*)) denotes the sub-tree rooted at sensor node *i. V*(●) denotes the set of sensor nodes on the tree; *E*(●) represents the set of edges of the tree, and *ζ_e_* is the length of edge *e*. The first item of *w*(*i*) represents the energy that has been consumed, and the second item denotes the energy that will be consumed in the sub-tree. Clearly, the root of the shortest path tree has the largest energy consumption, denoting the weight as *w*(*u*), among the sensor nodes on the tree.

When there are *k* actuators in the network, the network is divided into *k* regions. IPA-MMA first finds the farthest sensor node, *i*, on the shortest path tree, *T*, with the minimum weight. The basic idea of IPA-MMA is to iteratively find a sub-region for every actuator based on the sensor nodes' weights. Assume there are *k′* actuators left in each iteration. If *w*(*i*) < *w*(*u*)/*k′*, let *i* = *Pa* (*i*), where *Pa* (*i*) denotes the parent node of *i*. The process is repeated until *w*(*i*) > *w*(*u*)/*k′*. Then, the sensor node, *i*, is elected as the root of the sub-tree, and all the sensor nodes on the sub-tree will be associated with an actuator. At the same time, this sub-tree is removed from *T*. After that, update *w*(*u*), *k′* and *w*(*i*) for the left tree. Repeat the algorithm until there is only one actuator left. After the network has been successfully divided into *k* regions, use IPA to determine the tour for each actuator.

## 5. Performance Evaluation

In this section, several simulations have been conducted to compare and evaluate the behavior of our approach. The first group of simulations focuses on evaluating the network lifetime of three algorithms (IPA, SPT-DGA (data gathering algorithm) [18] and RD-VT (rendezvous design for variable tracks) [26]).

SPT-DGA is the shortest path tree based data gathering algorithm. The basic idea of SPT-DGA is to iteratively find a polling point among the sensor nodes on a shortest path tree, which is the nearest sensor to the root that can connect the remote sensors on the tree. Additionally, each polling point strives to link as many sensor nodes as it can reach within the relay hop bound to minimize the total number of polling points. RD-VT is the rendezvous design for variable tracks algorithm, for which the basic idea is to find a sub-tree, such that all the polling points on the sub-tree can be visited by a BStour no longer than *L*, while the total edge length of the path tree is minimized.

SPT-DGA and RD-VT do not consider the sensor nodes' residual energy, so the generated path tree is not dynamically updated; consequently, the selected polling points remain unchanged. However, in the proposed algorithm, IPA, the sensor nodes' residual energy is incorporated into the generated path tree; so, the path tree updates dynamically, and every round, new points would be selected as the polling points.

In the worst case scenario, the time complexity of SPT-DGA is *O*(*N*^2^ +*Nd*). The time complexity of RD-VT is *O*(*N*(log *N*)*^o^*^(^*^b^*^)^), for which *b* is an approximation constant with *b* > 1, and the complexity of the proposed algorithm IPA is *O*(*m*(*N*^2^ + *N* log *L*)). It is obvious that IPA is a little more complex than SPT-DGA and RD-VT. However, the greater computation expense could be justified by its increased network lifetime performance, as shown in the simulation.

### 5.1. Setting of Simulation Parameters

In this section, the simulation scene is constructed, and the parameters are set. There are *N* sensor nodes, organized in a random topology and randomly deployed in a square region. In Table 2, there are eight types of scenario sizes and seventeen kinds of sensor node numbers in total. The other parameters used in the simulations are set as follows. The data sink is located at the center of the network. The active time of the duty cycle is set to double of the data collection time constraint. The sensor node transmission range is 23 m. Each packet sent by a sensor node has a size of 100 bytes. The transfer rate is 250 kbps, and the moving velocity of the actuators is 1 m/s. In IPA, the topology of the network updates every five rounds. Each performance point is the average of the results of 100 simulation experiments.

In the simulations, the network lifetime performance and energy consumption performance are compared among the above algorithms, IPA, SPT-DGA and RD-VT. By adjusting the simulation network scale, the flexibility of the proposed algorithm, IPA, is demonstrated. Using two groups of 3D figures, the energy uniformity features of these algorithms are analyzed, showing that the main characteristic of the proposed algorithm is to balance the network sensor node energy consumption. In addition, the simulation of multiple actuators is implemented to verify the effectiveness of IPA-MMA.

### 5.2. Network Lifetime Performance

**Figure 4.**
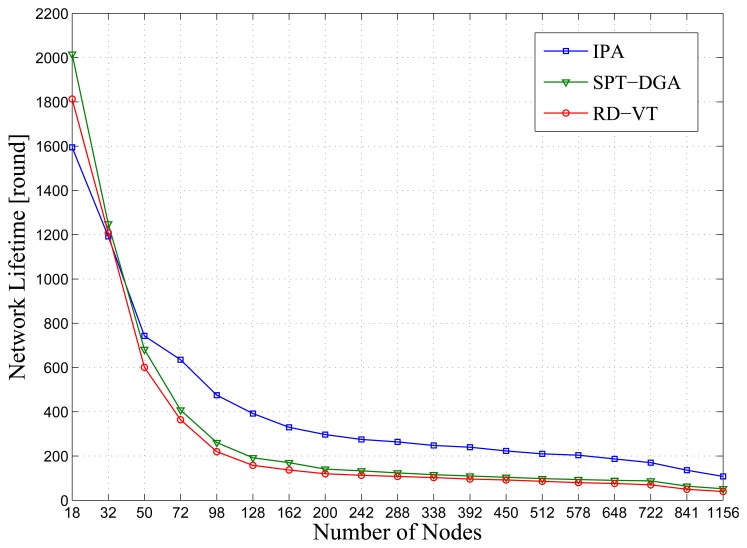
Network lifetime comparison for three algorithms. The *x*-axis is the scale of the network, and the *y*-axis is the network lifetime. SPT-DGA, shortest path tree based data gathering algorithm; RD-VT, rendezvous design for variable tracks.

In this section, we investigate the expected network lifetime for the sensor network model with different network scales. As shown in Figure 4, IPA achieves a significant performance gain over all other algorithms.

From the simulation results shown in Figure 4, we can draw the following conclusions. (1) As the number of sensor nodes in a network increases, the network lifetime decreases. It is obvious that as the number of sensor nodes increases, the sensor nodes close to the sink must transfer more data packets, consuming more energy; (2) When the number of sensor nodes, *N*, has increased beyond a certain threshold (*N* ⩾ 16), IPA will result in a higher network lifetime, because IPA generates routes and selects polling points in consideration of the sensor node's residual energy. When a network has fewer sensor nodes, it has a lower node distribution density, and the node distance tends to be larger, resulting in rapid energy consumption over a short period of time. Thus, IPA is better suited to larger scale networks.

### 5.3. Energy Consumption Performance

**Figure 5.**
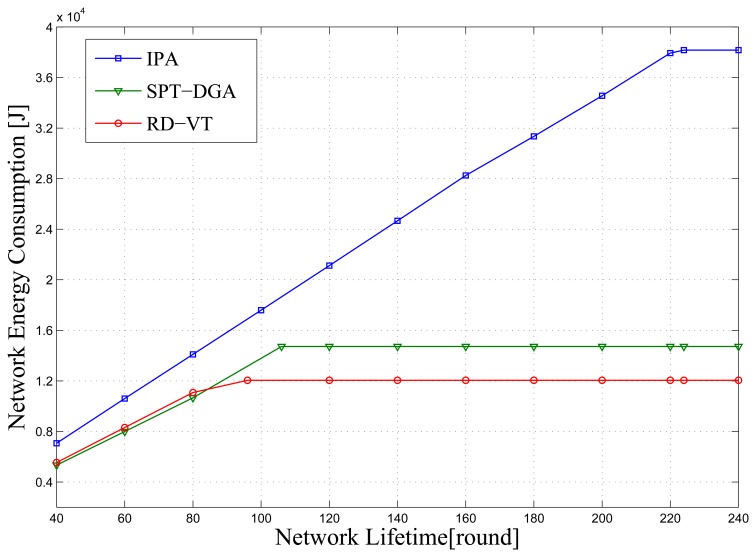
Network energy consumption comparison for three algorithms. The *x*-axis is the lifetime. IPA reconstructs the shortest path tree and then updates the polling point selection at the end of each round. The *y*-axis is the accumulation of network energy consumption. The lifetimes of IPA, SPT-DGA and RD-VT are 222 rounds, 104 rounds and 90 rounds, respectively.

In this section, we compare the three algorithms' network energy consumption over time, where the number of sensor nodes *N* = 450. Figure 5 shows the average result over 100 simulations, with the *x*-axis representing time in rounds and the *y*-axis representing the total network energy consumption. SPT-DGA and RD-VT expire at rounds 104 and 90, respectively (as shown in the figure by the energy consumption amount leveling off after those rounds), and IPA expires at round 222. It is clear that the IPA method has greater energy consumption during the network lifetime period. This high consumption is because IPA generates routes and selects polling points according to the residual energy of the sensor node; compared to the other two methods, IPA's routes result in a longer node communication distance, but increase the total network energy consumption.

### 5.4. Uniform Energy Consumption

**Figure 6.**
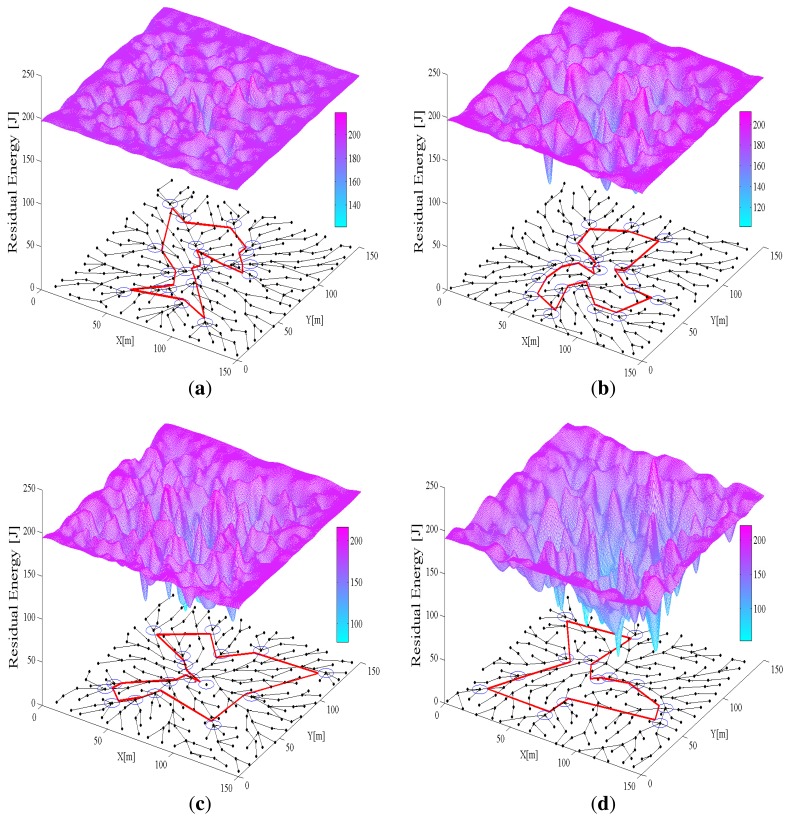
Distribution of network energy consumption with IPA. The *x* − *y* plane shows the initial network topology generated by IPA. The 3D surface is fit to the *x* − *y* − *z* data points (*x* − *y* is the 2D coordinate of a node; *z* is the residual energy of a node). (**a**–**d**) show the distribution of network energy consumption during the 10th, 50th, 90th and 104th rounds, respectively. (**a**) The distribution of network energy consumption with IPA during the 10th round; (**b**) the distribution of network energy consumption with IPA during the 50th round; (**c**) the distribution of network energy consumption with IPA during the 90th round; (d) the distribution of network energy consumption with IPA during the 104th round.

**Figure 7.**
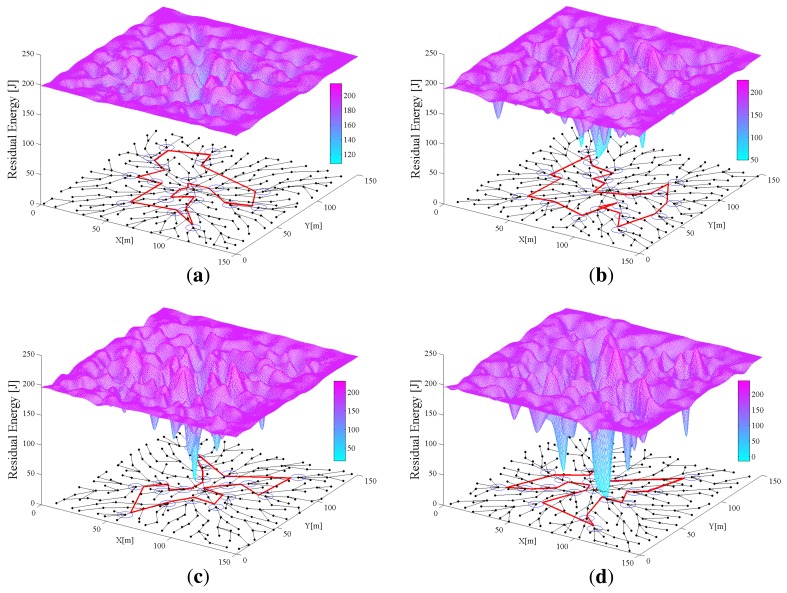
Distribution of network energy consumption with shortest path tree data gathering algorithm (SPT-DGA). The *x* − *y* plane shows the initial network topology generated by SPT-DGA. The 3D surface is fit to the *x* − *y* − *z* data points (*x* − *y* is the 2D coordinate of a node; *z* is the residual energy of a node). (**a**–**d**) show the distribution of network energy consumption during the 10th, 50th, 90th and 104th rounds, respectively (**a**) The distribution of network energy consumption with SPT-DGA during the 10th round; (**b**) the distribution of network energy consumption with SPT-DGA during the 50th round; (**c**) the distribution of network energy consumption with SPT-DGA during the 90th round; (**d**) the distribution of network energy consumption with SPT-DGA during the 104th round.

In this section, the performance of algorithms for uniform energy consumption is analyzed using two groups of 3D figures. In every 3D figure, the *x* − *y* plane shows the initial network topology generated by the above algorithms for each round and the *z*-axis is the residual energy of each sensor node in the network. Figures 6 and 7 show the simulation results of IPA and SPT-DGA, respectively, during rounds 10, 50, 90 and 104. Through Figures 6 and 7, we can observe the energy variation of each sensor node and compare the performance of the IPA and SPT-DGA algorithms in uniform energy consumption.

As shown in Figure 6, the traffic load is concentrated on the sensor nodes close to the data sink and distributed symmetrically on the sensor nodes surrounding it. The network lifetime is determined by these sensor nodes close to the data sink, because of their heavy load. The IPA algorithm will let these sensor nodes distribute the load more uniformly. The topology of the network and the moving path of the actuator change over time in the IPA algorithm. This dynamic character can improve the network's energy efficiency and extend the network's lifetime. Because SPT-DGA is a static algorithm, the sensor nodes close to the polling points of the actuator exhaust their energy more quickly, leading to non-uniform energy consumption and restricting the network's lifetime. By using 3D graphs, we can clearly see that the uniform energy consumption performance of IPA is better, which is the reason that IPA increases the network lifetime.

### 5.5. Energy Consumption Performance for IPA-MMA

**Figure 8.**
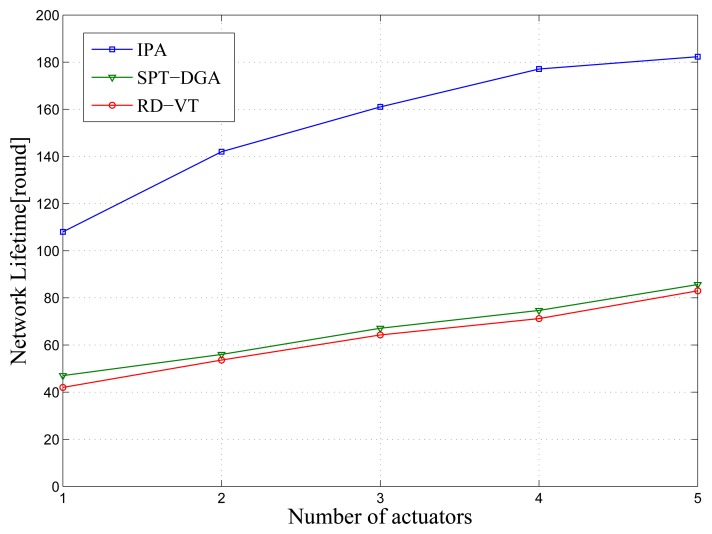
Energy consumption *versus* number of actuators.

In this section, the energy consumption performance for the multi-actuator case where the number of sensor nodes, *N*, is set to 1,156 is compared. In the multi-actuator case, it is critical to partition the actuator region correctly. As shown in Figure 8, IPA-MMA achieves a significant performance gain over all other algorithms, such as power savings, shorter latency, and so on. The main reasons are as follows:

(1) IPA-MMA takes the sensor node's residual energy as one of the division parameters. Firstly, IPA-MMA constructs the shortest path tree using Equation (20). Then, each sensor node will be assigned a weight calculated by Equation (21), which includes the residual energy and the distance to its child sensor nodes. According to their weight, each sensor node decides their affiliated sub-region traveled by one actuator, and finally, the sum weights of these divisions achieve a balance. In this case, sensor nodes with more residual energy have more opportunity to relay others' data. This will produce more uniform energy consumption and more total lifetime length of network than the distance-based division.
(2) IPA-MMA divides the sensing fields dynamically. The compared algorithms, SPT-DGA and RD-VT, with fixed divisions, will result in more energy consumption for center nodes, and energy holes are also created. IPA-MMA updates its division after certain rounds according to the new residual energy distribution and balances the weight of each division again. Thus, more uniform energy consumption can be achieved than the compared algorithms.

## 6. Conclusions

This paper has suggested a path-planning algorithm for the actuator in a wireless sensor-actuator network to collect data in delay-constrained real-time applications, especially for large-scale networks. In this algorithm, the shortest path tree topology is dynamically reconstructed using the residual energies in the sensor nodes as the weights based on an integrated energy model, which considers energy consumed during communications, node sensing and data processing, describing the energy consumption more closely to actual circumstances. Polling points are chosen for each round based on the in-degrees of sensor nodes in the shortest path tree. Additionally, the new polling point selection method takes into consideration the actuator communication range, so that the actuator can visit more polling points, while under the same time constraint, and increase network lifetime. The energy efficiency of large networks is thus raised at the cost of a slight increase in algorithm complexity. In order to address the application for multiple mobile actuators, an IPA-MMA algorithm is also proposed to adapt to large-scale networks. The simulation results show that the network lifetime is greatly extended at the cost of moderately increasing the total energy consumption of the network.

In the future, the variation of data transmission frequencies and the limitations of sensor node data buffers can also be incorporated into the algorithm design. Additionally, more delicate techniques based on convex optimization might be adopted to address the data collection problem when integrating these factors more flexibly.

## Figures and Tables

**Table 1.** Notations used.

**Variables**	**Means**
*A_k_*	the sensor node set of the region assigned to actuator k;
*y_iu_* = {0, 1} ∀*i*, *u* ∈ *V*	if sensor node *i* is rooted on sensor node *u*, *y_iu_* = 1, otherwise zero;
*I_u_* = {0, 1} ∀*u* ∈ *V*	if sensor node *u* is chosen as the polling point, *I_u_* = 1, otherwise zero;
*x_iju_* = {0, 1} ∀*i*, *j*, *u* ∈ *V*	if the data of sensor node *i* forwarded by sensor node *j* and rooted on sensor node *u*, *x_iju_* = 1, otherwise zero;
*n_ij_* = {0,1} ∀*i*,*j* ∈ *V*	if sensor node *i* is a neighbor of sensor node *j*, *n_ij_* = 1, otherwise zero;
*z_uk_* = {0, 1} ∀*u* ∈ *V*, ∀*k* ∈ *κ*	if sensor node *u* is visited by actuator *k*, *z_uk_* = 1, otherwise zero;
*e_pqk_* = {0, 1} ∀*p*, *q* ∈ *V*, ∀*k* ∈ *κ*	if link (*p*, *q*) is visited by actuator *k*,*e_pqk_* = 1, otherwise zero;
*l_pq_*, *C_ij_* ∀*p*, *q*, *i*, *j* ∈ *V*	the length of link (*p*, *q*) and the cost of link (*i*, *j*).
*E_init_*	the initial energy of the sensor node.

**Table 2.** Simulation parameters of different network scales.

**Number of Nodes**	**Network Size** (**m** × **m**)	**Maximum Moving Distance of Actuators in a Round (m)**
18	30 × 30	20
32/50	40 × 40	30
72/98/128	70 × 70	100
162/200/242	100 × 100	200
288/338/392/450/512	150 × 150	450
578/648/722	200 × 200	700
841	500 × 500	850
1,156	1,000 × 1,000	1,000
